# Predicting early acute pancreatitis severity using WBC, neutrophil count, NLR, and glucose levels

**DOI:** 10.1097/MD.0000000000050587

**Published:** 2026-09-11

**Authors:** Zhongshan Li, Yue Lou, Guangfeng Zhang, Xuan Fang, Shiming Chen

**Affiliations:** a Department of Hepatobiliary Pancreatic Surgery, Shaoxing Second Hospital, Shaoxing, Zhejiang, P.R. China.

**Keywords:** acute pancreatitis, glucose, neutrophil, neutrophil-to-lymphocyte ratio, white blood cell

## Abstract

Early prediction of acute pancreatitis (AP) severity is critical for improving outcomes. This study evaluated the diagnostic value of white blood cell (WBC) count, neutrophil count, neutrophil-to-lymphocyte ratio (NLR), and glucose level for early severity prediction. In this retrospective cohort study, 192 patients with AP (March 2019–December 2021) were stratified by severity using the Revised Atlanta Classification. Blood samples were collected at admission. Demographic and biochemical data were analyzed to identify factors associated with moderately severe (M-SAP) or severe (SAP) AP. Of 192 patients, 61 progressed to M-SAP/SAP. Multivariate analysis identified WBC count, neutrophil count, NLR, and blood glucose as independent predictors for M-SAP/SAP in biliary or alcoholic AP (all *P* < .05). Receiver operating characteristic curve analysis demonstrated significant predictive value for each individual biomarker. Notably, NLR showed superior predictive accuracy in alcoholic AP (area under the curve [AUC] = 0.828) compared with WBC count (AUC = 0.653) and glucose (AUC = 0.675), whereas WBC count demonstrated higher specificity (84.5%) in biliary AP. WBC count, neutrophil count, NLR, and blood glucose are valuable, independent predictors for early progression to M-SAP/SAP in biliary or alcoholic AP, with each marker reflecting distinct aspects of the inflammatory and metabolic response. A composite model integrating these factors provides enhanced predictive performance (AUC = 0.89). In clinical practice, this simple, cost-effective panel can stratify patients by risk at admission, guide triage decisions, and facilitate early intensive monitoring for high-risk individuals, thereby potentially improving outcomes through timely intervention.

## 1. Introduction

Acute pancreatitis (AP) is a common acute abdominal condition, and its incidence appears to be increasing. AP is usually regarded as a mild and self-limiting illness with minimal systemic effects and good outcomes.^[1]^ However, approximately 20% to 30% of all patients with AP encountered in clinical practice eventually develop SAP, experiencing a severe attack with progression to multiorgan dysfunction or local complications.^[2]^ Despite improvements in diagnostic and therapeutic techniques, the mortality rate of SAP is relatively high, ranging from 20% to 40%, with no decreasing trend observed over the last decade.^[3]^ Early recognition of patients with severe disease has been shown to improve the prognosis and reduce mortality by facilitating close monitoring, early intensive therapy, and proper timing of interventions.

Currently, a multitude of clinical assessment tools exist for the evaluation of severe pancreatitis, encompassing clinical examination, computed tomography (CT) imaging, a spectrum of scoring systems, and laboratory parameter measurement.^[4,5]^ Despite this diversity, a consensus on a standardized and precise diagnostic criterion for severe pancreatitis remains elusive.^[6]^

Four widely recognized scoring systems are currently employed by clinicians to aid them in distinguishing severe AP (SAP) and moderately severe AP (M-SAP): the Acute Physiology and Chronic Health Evaluation II (APACHE-II), Ranson, Bedside Index of Severity in Acute Pancreatitis, and modified computed tomography severity index (MCTSI) scoring systems. Each system offers unique applications and benefits, yet they all have inherent limitations. The APACHE-II and Ranson scoring systems are particularly valuable for their diagnostic significance. However, use of the Ranson scoring system, which requires a 48-hour period for completion, may delay crucial treatment decisions.^[7,8]^ On the other hand, the APACHE-II scoring system, while comprehensive, is often criticized for its complexity and the time investment needed to gather numerous required indicators, some of which may not be readily available in standard ward settings.^[9–11]^ The Bedside Index of Severity in Acute Pancreatitis scoring system is favored for its simplicity and ease of use, but it falls short in differentiating between patients with temporary and prolonged organ dysfunction, potentially leading to an overestimation of severity.^[11]^ Finally, the MCTSI scoring system necessitates contrast-enhanced CT imaging, which, according to current domestic and international guidelines, if conducted within 72 hours of symptom onset, may underestimate or misclassify the severity of the disease. This timing issue renders the MCTSI scoring system less effective for early prediction of AP.^[12,13]^ Consequently, conventional scoring systems present challenges in accurately predicting the early onset of SAP in patients, highlighting the need for more efficient and timely predictive tools.

Accordingly, the present study focused on examining the associations between various inflammatory and clinical parameters in AP patients who experienced the onset of abdominal pain within a 24-hour period. Specifically, we analyzed indicators such as the white blood cell (WBC) count, neutrophil count, neutrophil-to-lymphocyte ratio (NLR), and blood glucose level. The study aimed to elucidate the relationships between these markers and the severity or progression of AP.

## 2. Materials and methods

### 2.1. Patient selection

**Figure 1.**
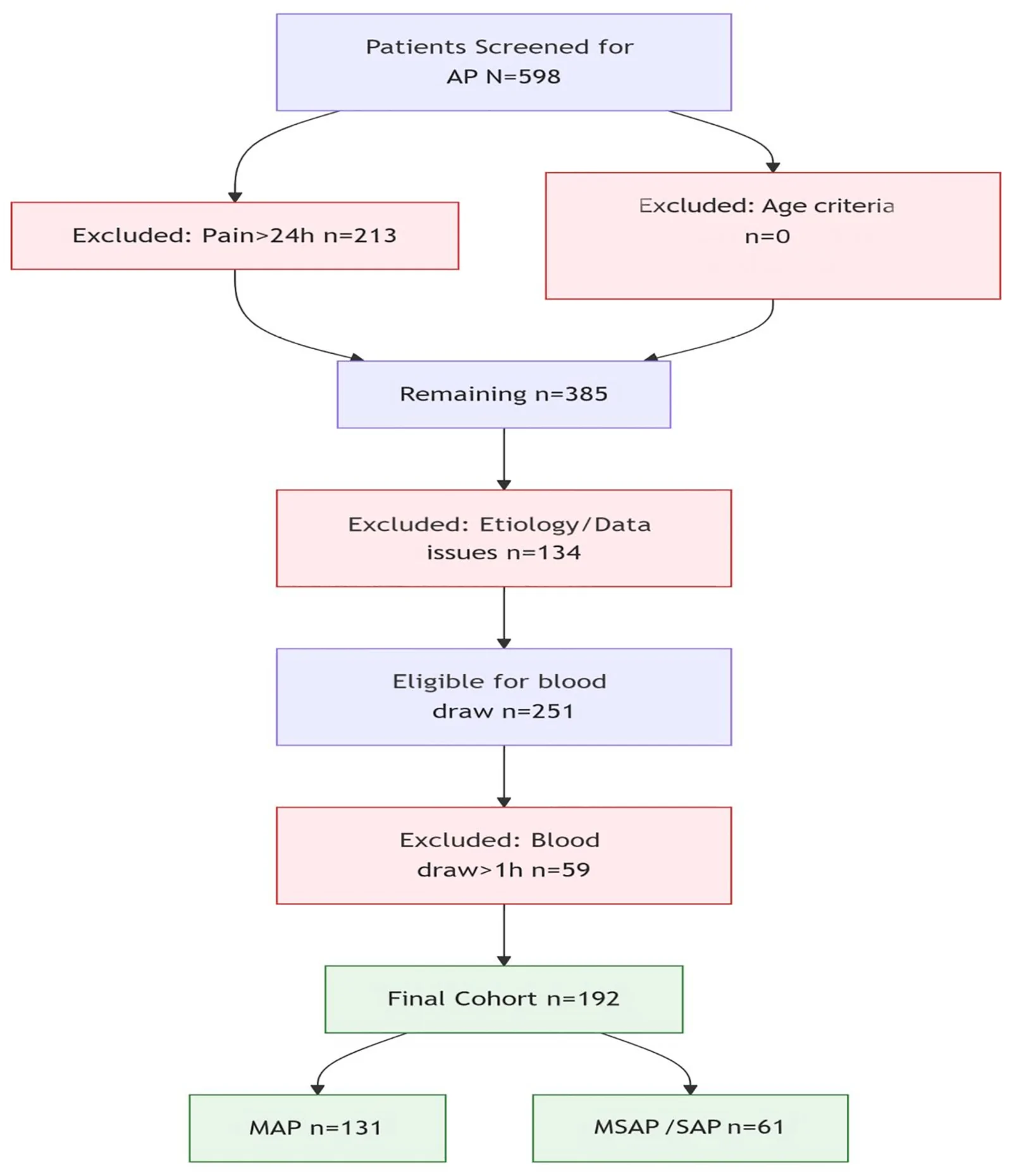
Flow diagram of patient population. AP = acute pancreatitis, M-SAP = moderately severe acute pancreatitis, SAP = severe acute pancreatitis.

We retrospectively analyzed patients who were admitted to the Department of Hepatobiliary and Pancreatic Surgery, the Second Affiliated Hospital of Shaoxing University (Shaoxing, China), with a confirmed diagnosis of AP between March 2019 and September 2021. Individuals were excluded if they met any of the following criteria: age <18 years or >80 years, time from symptom onset to hospital admission >24 hours, patients with blood samples drawn >1 hour after admission, pancreatitis induced by trauma or pregnancy, post-endoscopic retrograde cholangiopancreatography pancreatitis, chronic pancreatitis, a history of malignant tumor or hemoproliferative disorder, recent chemotherapy or treatment with steroids or antibiotics, recent blood transfusion, or unavailable laboratory measurements or medical records. After exclusion, the total number of eligible patients for this study was 192 (Fig. 1). For this study, informed consent was waived by the Second Affiliated Hospital of Shaoxing University Institutional Review Board/ethics committee due to the anonymity and retrospective nature of the study.

### 2.2. Diagnostic criteria

According to the 2012 International Association of Pancreatology report, “Atlanta Classification and Definitions of AP (revised),”^[14]^ AP was defined as clinical findings based on the presence of 2 or more of the following conditions: abdominal pain consistent with AP; serum amylase and/or lipase level elevation ≥3 times the upper limit of normal value; and characteristic manifestations of AP on contrast-enhanced CT, magnetic resonance imaging, or transabdominal ultrasonography. M-SAP was characterized by organ failure (OF) that resolves within 48 hours (transient OF) and/or local or systemic complications without persistent organ failure (POF). SAP was defined as AP with persistent OF (>48 hours). POF was diagnosed when the score for one or more of the following modified Marshall scoring system criteria was ≥2: respiratory failure if the PaO_2_/FiO_2_ was <300 mm Hg; renal failure if the serum creatinine level was ≥1.9 mg/dL; and cardiovascular failure if the systolic blood pressure was <90 mm Hg despite fluid replacement. The presence of acute peripancreatic fluid accumulation, acute necrosis accumulation, pancreatic pseudocysts, or parenchymal necrosis was required for the diagnosis of local complications.

This study combined M-SAP and SAP into a single group for analysis, primarily on the basis of the following considerations. First, although the 2012 Revised Atlanta Classification suggests similarities between M-SAP and mild acute pancreatitis (MAP) in certain aspects, clinical practice evidence demonstrates that M-SAP patients may still face complications and uncertainties in terms of disease progression. Second, owing to the limited sample size, merging M-SAP and SAP patients increased the sample size to better interpret data trends and differences. Additionally, due to the limited sample size of patients with SAP (n = 15), subgroup analysis comparing M-SAP and SAP was not performed to avoid small-sample bias and potential false-negative or false-positive results.

### 2.3. Clinical data collection

The patients’ medical records were reviewed, and the following information was collected: demographic information (age, sex, and etiology), medical history (hypertension, diabetes mellitus, and liver cirrhosis), smoking habits, alcohol consumption, preexisting comorbidities, intensive care unit admission, duration of hospital stay, and mortality. Laboratory data included WBC, neutrophil, lymphocyte, and platelet counts; NLR; platelet-to-lymphocyte ratio (PLR); hematocrit; and calcium, high-sensitivity C-reactive protein, blood urea nitrogen, albumin, glucose, lactate dehydrogenase (LDH), aspartate aminotransferase, alanine aminotransferase, and blood amylase levels, as well as the Ranson score. Blood samples were collected within 1 hour after hospitalization and then analyzed with an automated medical analyzer within 6 hours of collection in the core clinical laboratory of our hospital. The NLR and PLR were defined as the quotient of the absolute neutrophil count to the absolute lymphocyte count and that of the absolute platelet count to the absolute lymphocyte count, respectively.

### 2.4. Data analysis

Data analysis was performed with SPSS 20.0 (SPSS Inc.). Continuous variables with a normal distribution are presented as the mean ± standard deviation, and Student *t* test was used to compare 2 groups. If variables were nonnormally distributed, data are shown as the median (25th–75th percentile), and a nonparametric Mann–Whitney *U* test was chosen to compare 2 groups. Categorical variables were compared using a chi-square test or Fisher exact test. To assess the correlations between the NLR and the severity of AP and the Ranson score, Spearman correlation analysis was performed. The optimal cutoff value of the NLR was estimated via receiver operating characteristic (ROC) curve analysis, and the accuracy of the predictive power of the NLR was determined by the area under the ROC (AUC). *P* values < .05 were considered statistically significant.

### 2.5. Ethics and consent to participate

This research was performed in accordance with the principles of the Declaration of Helsinki and was approved by the Ethics Committee of Shaoxing Second Hospital. The requirement to obtain informed consent from individuals was waived because patients’ medical records and information were anonymously collected before analysis.

## 3. Results

### 3.1. Patient characteristics

The baseline characteristics of the 192 patients with AP included in our study (males: 100, 52.1%; females: 92, 47.9%) are presented in Table 1. Among these 192 patients, 61 were in the M-SAP/SAP group. The mean age was 56.76 ± 14.70 years. The most common type of AP was biliary AP (n = 117, 60.9%), followed by hypertriglyceridemia AP (n = 35, 18.2%), alcoholic AP (n = 30, 15.6%), and idiopathic AP (n = 10, 5.2%). There was no significant difference between the MAP group and the M-SAP/SAP group in terms of sex; age; etiology; lymphocyte or platelet count; PLR; hematocrit; or calcium, high-sensitivity C-reactive protein, aspartate aminotransferase, alanine aminotransferase, or blood amylase level (all *P *> .05). The WBC count, neutrophil count, NLR, glucose level, LDH level, and Ranson score were significantly greater in the M-SAP/SAP group than in the MAP group (*P *< .05; Table 1).

**Table 1:** Baseline characteristics of patients with AP.

Variable	All patients	MAP (n = 131)	M-SAP/SAP (n = 61)	*P* value
Sex (male, %)	103 (53.4)	66 (50.4)	35 (57.3)	.366
Age (yr)	56.76 ± 14.70	55.30 ± 15.52	57.34 ± 14.32	.387
Etiology (%)				.068
Gallstones	117 (60.9)	84 (43.8)	33 (17.2)	
Hypertriglyceridemia AP	35 (18.2)	18 (9.4)	17 (8.9)	
Alcoholic AP	30 (15.6)	19 (9.9)	11 (5.7)	
Idiopathic AP	10 (5.2)	6 (3.1)	4 (2.1)	
Laboratory parameters (at admission)				
WBC count (×10^9^/L)	11.44 ± 4.29	10.53 ± 3.23	13.40 ± 5.49	<.001
Neutrophil count (×10^9^/L)	9.41 ± 4.14	8.53 ± 3.21	11.30 ± 5.18	<.001
Lymphocyte count (×10^9^/L)	1.44 ± 0.99	1.49 ± 1.07	1.33 ± 0.82	.148
Platelet count (×10^9^/L)	198.95 ± 58.47	194.64 ± 54.06	208.21 ± 66.50	.197
NLR	9.18 ± 8.10	8.12 ± 7.66	11.47 ± 8.60	.002
PLR	186.36 ± 124.00	177.11 ± 119.82	206.22 ± 131.35	.14
Hct (%)	0.42 ± 0.05	0.41 ± 0.05	0.42 ± 0.07	.188
Calcium (mmol/L)	2.20 ± 0.24	2.22 ± 0.23	2.17 ± 0.27	.273
Hs-CRP (mg/L), median (P_25_–P_75_)	7.15 (2.00–32.83)	7.70 (1.80–22.90)	7.00 (2.95–86.15)	.307
BUN (mmol/L), median (P_25_–P_75_)	4.90 (3.80–6.10)	4.90 (3.70–6.15)	4.96 (3.90–6.10)	.942
Albumin (g/L)	37.76 ± 4.58	37.92 ± 4.51	37.41 ± 4.76	.441
Glucose (mmol/L)	7.98 ± 4.09	7.37 ± 2.79	9.29 ± 5.82	.002
LDH (U/L), median (P_25_–P_75_)	239.5 (192.5–330.5)	226.0 (184.0–312.0)	292.0 (214.0–3653.5)	.006
AST (U/L), median (P_25_–P_75_)	63.0 (23.25–206.75)	59.0 (22.00–194.0)	85.0 (27.00–218.50)	.466
ALT (U/L), median (P_25_–P_75_)	59.5 (22.0–214.8)	59.0 (20.0–214.0)	66.0 (26.5–251.0)	.704
Blood amylase (U/L), median (P_25_–P_75_)	619.35 (206.7–1492.8)	612.00 (204.9–1409.8)	821.00 (217.0–2104.1)	.152
Ranson score	2.10 ± 1.29	1.89 ± 0.10	2.57 ± 0.18	<.001

The data are expressed as the means ± SDs or as medians (P_25_–P_75_).

ALT = alanine aminotransferase, AP = acute pancreatitis, AST = aspartate aminotransferase, BUN = blood urea nitrogen, Hct = hematocrit, Hs-CRP = high-sensitivity C-reactive protein, LDH = lactate dehydrogenase, MAP = mild acute pancreatitis, M-SAP = moderately severe acute pancreatitis, NLR = neutrophil-to-lymphocyte ratio, PLR = platelet-to-lymphocyte ratio, SAP = severe acute pancreatitis, SD = standard deviation, WBC = white blood cell.

*P* < .05.

### 3.2. Analysis of factors affecting M-SAP/SAP

The factors found to be statistically significant in the univariate analysis were further analyzed by multivariate logistic regression analysis, which revealed that the WBC count (odds ratio [OR] = 1.177, *P* < .001, 95% confidence interval [CI] = 1.088–1.274), neutrophil count (OR = 1.185, *P* < .001, 95% CI = 1.091–1.287), NLR (OR = 1.053, *P* = .016, 95% CI = 1.010–1.099), glucose level (OR = 1.154, *P* = .006, 95% CI = 1.041–1.279) and Ranson score (OR = 1.534, *P* = .001, 95% CI = 1.192–1.973) were independent factors associated with the risk of M-SAP/SAP (Table 2).

**Table 2:** Multivariate logistic regression analysis of M-SAP/SAP.

Variable	B	SE	Wals	*P* value	OR	95% CI
WBC count (×10^9^/L)	0.163	0.040	16.308	<.001	1.177	1.088–1.274
Neutrophil count (×10^9^/L)	0.169	0.042	16.236	<.001	1.185	1.091–1.287
NLR	0.052	0.022	5.852	.016	1.053	1.010–1.099
Glucose (mmol/L)	0.143	0.052	7.474	.006	1.154	1.041–1.279
Ranson score	0.428	0.129	11.081	.001	1.534	1.192–1.973

M-SAP = moderately severe acute pancreatitis, NLR = neutrophil-to-lymphocyte ratio, SAP = severe acute pancreatitis, SE = standard error, WBC = white blood cell, OR = odds ratio, CI = confidence interval.

*P* < .05.

### 3.3. Correlations of the WBC count, neutrophil count, NLR, and glucose level with the Ranson score in M-SAP/SAP patients

**Figure 2.**
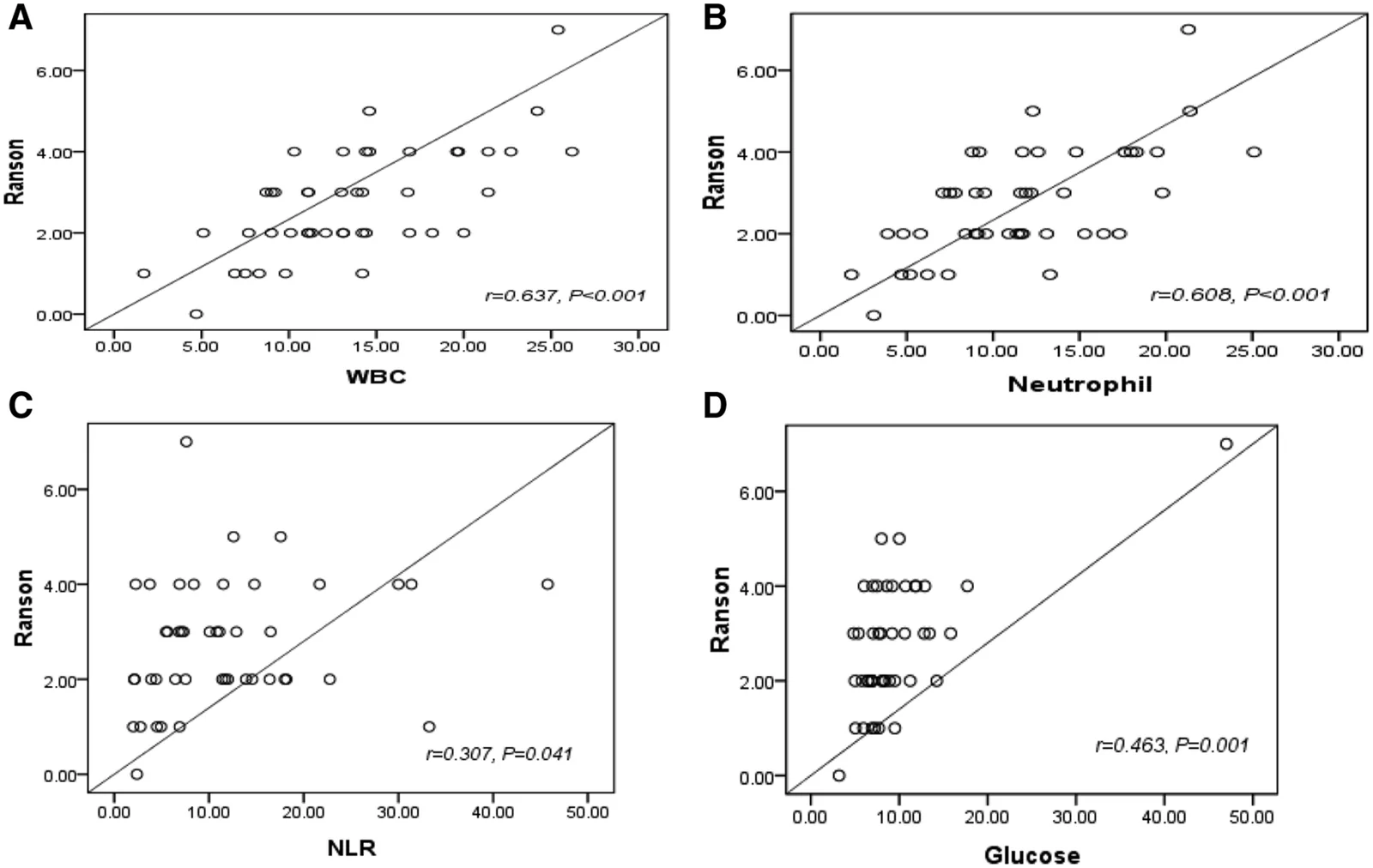
Correlations of the WBC count (A) with the Ranson score. Correlations of the neutrophil count (B) with the Ranson score. Correlations of the NLR (C) with the Ranson score. Correlations of the glucose level (D) with the Ranson score. NLR = neutrophil-to-lymphocyte ratio, WBC = white blood cell.

Spearman correlation analysis revealed that the WBC count, neutrophil count, NLR, and glucose level were positively correlated with the Ranson score (*r* = 0.637, *P *< .001; *r* = 0.608, *P *< .001; *r* = 0.307, *P *= .041; *r* = 0.463, *P = *.001, respectively; Fig. 2A–D), suggesting that these factors are markers for M-SAP/SAP evaluation.

### 3.4. Stratified analysis of factors according to AP etiology

We performed subgroup analysis according to AP etiology. For biliary AP, the mean WBC count, neutrophil count, NLR, and glucose level in the M-SAP/SAP group were significantly greater than those in the MAP group (13.44 ± 5.60 vs 9.92 ± 2.99, 11.30 ± 5.41 vs 8.01 ± 3.04, 10.82 [4.74–14.90] vs 6.10 [4.16–10.11], and 8.40 ± 2.93 vs 6.97 ± 2.17, *P* < .05, respectively; Table 3). For hypertriglyceridemia AP (HTG-AP), although the NLR and glucose level were greater in the M-SAP/SAP group than in the MAP group, there were no significant differences between the 2 groups (8.37 ± 5.61 vs 6.37 ± 3.38 and 9.26 ± 3.85 vs 8.60 ± 3.60, *P *> .05, respectively; Table 4). Similarly, there were no differences between the groups in terms of WBC or neutrophil counts (12.59 ± 4.65 vs 12.86 ± 3.26, 10.44 ± 4.49 vs 10.54 ± 3.33, *P *> .05; Table 4). For alcoholic AP, the mean WBC count, neutrophil count, NLR, and glucose level in the M-SAP/SAP group were significantly greater than those in the MAP group (14.56 ± 6.58 vs 10.86 ± 3.28, 12.65 ± 5.64 vs 8.81 ± 3.20, 14.11 ± 6.43 vs 7.64 ± 3.36, and 11.78 ± 4.80 vs 6.89 ± 1.95, *P* < .05, respectively; Table 5).

**Table 3:** Comparison of the WBC count, neutrophil count, NLR, and glucose level in biliary AP patients.

Variable	MAP	M-SAP/SAP	*t*/*Z*	*P* value	95% CI
WBC count (×10^9^/L)	9.92 ± 2.99	13.44 ± 5.60	4.396	<.001	1.933–5.104
Neutrophil count (×10^9^/L)	8.01 ± 3.04	11.30 ± 5.41	4.168	<.001	1.729–4.959
NLR, median (P_25_–P_75_)	6.10 (4.16–10.11)	10.82 (4.74–14.90)	−2.253	.024	–
Glucose (mmol/L)	6.97 ± 2.17	8.40 ± 2.93	2.892	.005	0.451–2.413

AP = acute pancreatitis, CI = confidence interval, MAP = mild acute pancreatitis, M-SAP = moderately severe acute pancreatitis, NLR = neutrophil-to-lymphocyte ratio, SAP = severe acute pancreatitis, WBC = white blood cell.

*P* < .05.

**Table 4:** Comparison of the WBC count, neutrophil count, NLR, and glucose level in hypertriglyceridemia AP patients.

Variable	MAP	M-SAP/SAP	*t*/*Z*	*P* value	95% CI
WBC count (×10^9^/L)	12.86 ± 3.26	12.59 ± 4.65	−0.194	.848	−3.010 to 2.487
Neutrophil count (×10^9^/L)	10.54 ± 3.33	10.44 ± 4.49	−0.078	.938	−2.813 to 2.606
NLR	6.37 ± 3.38	8.37 ± 5.61	1.288	.207	−1.161 to 5.166
Glucose (mmol/L)	8.60 ± 3.60	9.26 ± 3.85	0.526	.602	−1.899 to 3.224

AP = acute pancreatitis, CI = confidence interval, MAP = mild acute pancreatitis, M-SAP = moderately severe acute pancreatitis, NLR = neutrophil-to-lymphocyte ratio, SAP = severe acute pancreatitis, WBC = white blood cell.

*P* > .05.

**Table 5:** Comparison of the WBC count, neutrophil count, NLR, and glucose level in alcoholic AP patients.

Variable	MAP	M-SAP/SAP	*t*/*Z*	*P* value	95% CI
WBC count (×10^9^/L)	10.86 ± 3.28	14.56 ± 6.58	2.377	.023	0.548 to 6.850
Neutrophil count (×10^9^/L)	8.81 ± 3.20	12.65 ± 5.64	2.719	.010	0.982 to 6.707
NLR	7.64 ± 3.36	14.11 ± 6.43	3.118	.003	2.268 to 10.660
Glucose (mmol/L)	6.89 ± 1.95	11.78 ± 4.80	1.929	.045	−0.464 to 10.176

AP = acute pancreatitis, CI = confidence interval, MAP = mild acute pancreatitis, M-SAP = moderately severe acute pancreatitis, NLR = neutrophil-to-lymphocyte ratio, SAP = severe acute pancreatitis, WBC = white blood cell.

*P* < .05.

### 3.5. Predictive value of the WBC count, neutrophil count, NLR, and glucose level in predicting M-SAP/SAP development in patients with biliary AP and alcoholic AP

**Figure 3.**
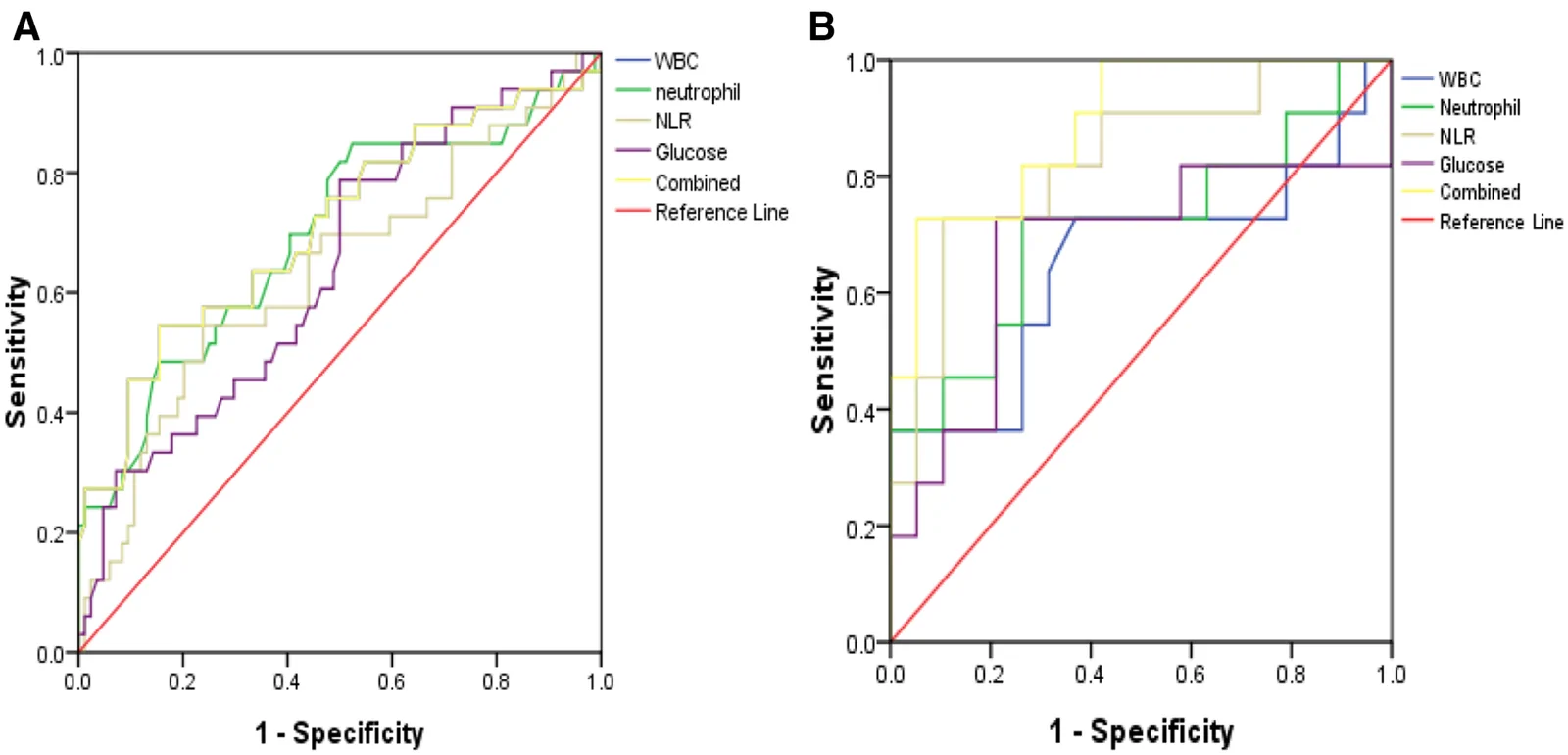
ROC curves for predicting M-SAP/SAP. (A) Biliary AP. ROC curves for predicting M-SAP/SAP. (B) Alcoholic AP. AP = acute pancreatitis, M-SAP = moderately severe acute pancreatitis, NLR = neutrophil-to-lymphocyte ratio, ROC = receiver operating characteristic, SAP = severe acute pancreatitis, WBC = white blood cell.

ROC curves of the performance of the WBC count, neutrophil count, NLR, and glucose level measured on admission, which showed obvious statistical significance in biliary AP and alcoholic AP, in predicting M-SAP/SAP, were plotted. For biliary AP (Table 6 and Fig. 3A), the AUC, cutoff, sensitivity, and specificity of the WBC count were 0.707, 12.95 × 10^9^/L, 54.5%, and 84.5%, respectively; those of the neutrophil count were 0.693, 11.25 × 10^9^/L, 48.5%, and 84.5%, respectively; those of the NLR were 0.634, 10.45, 54.5%, and 76.2%, respectively; and those of the glucose level were 0.641, 6.8 mmol/L, 50%, and 50%, respectively. For alcoholic AP (Table 7 and Fig. 3B), the AUC, cutoff, sensitivity, and specificity of the WBC count were 0.653, 17.85 × 10^9^/L, 36.4%, and 100%, respectively; those of the neutrophil count were 0.713, 10.8 × 10^9^/L, 72.7%, and 73.7%, respectively; those of the NLR were 0.828, 10.42, 72.7%, and 89.5%, respectively; and those of the glucose level were 0.675, 7.53 mmol/L, 72.7%, and 94.7%, respectively. In the 2 AP groups, the AUC values of the combined model (0.787 and 0.89, respectively) were greater than those of any individual factor, indicating that the combination of the WBC count, neutrophil count, NLR, and glucose level can serve as a panel of predictive factors in patients with AP and that the performance of this combination was superior to that of any individual factor. Notably, the predictive performance of individual biomarkers differed by etiology. In alcoholic AP, the NLR demonstrated superior discriminatory ability (AUC = 0.828) compared with WBC count (AUC = 0.653) and glucose level (AUC = 0.675), suggesting that the neutrophil-to-lymphocyte balance may be a more sensitive indicator of the inflammatory response in this subgroup. In contrast, for biliary AP, all 4 markers exhibited more comparable predictive values, with WBC count showing the highest specificity (84.5%). These findings underscore the importance of etiology-specific interpretation of these routine biomarkers in clinical practice.

**Table 6:** Value of the WBC count, neutrophil count, NLR, and glucose level in predicting M-SAP/SAP development in patients with biliary AP.

Index	AUC	95% CI	*P* value	Cutoff	Sensitivity	Specificity
WBC count (×10^9^/L)	0.707	0.595–0.820	<.001	12.95	54.5%	84.5%
Neutrophil count (×10^9^/L)	0.693	0.580–0.807	.001	11.25	48.5%	84.5%
NLR	0.634	0.516–0.752	.024	10.45	54.5%	76.2%
Glucose (mmol/L)	0.641	0.529–0.752	.018	6.80	50.0%	50.0%
WNNG panel	0.787	0.595–0.820	<.001	0.38	54.5%	84.5%

AP = acute pancreatitis, AUC = area under the curve, CI = confidence interval, M-SAP = moderately severe acute pancreatitis, NLR = neutrophil–lymphocyte ratio, SAP = severe acute pancreatitis, WBC = white blood cell, WNNG = WBC-neutrophil-NLR-glucose.

*P* < .05.

**Table 7:** Value of the WBC count, neutrophil count, NLR, and glucose level in predicting M-SAP/SAP development in patients with alcoholic AP.

Index	AUC	95% CI	*P* value	Cutoff	Sensitivity	Specificity
WBC count (×10^9^/L)	0.653	0.426–0.881	.048	17.85	36.4%	100%
Neutrophil count (×10^9^/L)	0.713	0.504–0.992	.015	10.80	72.7%	73.7%
NLR	0.828	0.669–0.986	.003	10.42	72.7%	89.5%
Glucose (mmol/L)	0.675	0.445–0.904	.036	7.53	72.7%	78.9%
WNNG panel	0.890	0.773–1.007	<.001	0.57	72.7%	94.7%

AP = acute pancreatitis, AUC = area under the curve, CI = confidence interval, M-SAP = moderately severe acute pancreatitis, NLR = neutrophil-to-lymphocyte ratio, SAP = severe acute pancreatitis, WBC = white blood cell, WNNG = WBC-neutrophil-NLR-glucose.

*P* < .05.

## 4. Discussion

The condition of patients with severe pancreatitis progresses rapidly, and finding a test that can accurately determine a patient’s condition in a timely manner is essential for clinical treatment and outcomes, especially on the day of admission, as this period is regarded as a window of opportunity to prevent pancreatic necrosis and POF, which is also the core of the treatment of clinical acute severe pancreatitis.^[1,15,16]^ Despite the importance of early intervention, the intricate and unpredictable nature of the disease course presents significant challenges in predicting severity at onset.^[4]^ Accurate early assessment is paramount to initiating appropriate therapeutic measures and potentially mitigating progression to severe pancreatitis.

In the initial phase of SAP, a cascade of inflammatory cytokines is released, leading to the release of various cytokines and inflammatory mediators. This complex interplay culminates in systemic inflammatory response syndrome, a critical hallmark of SAP.^[17,18]^ Consequently, inflammatory markers are pivotal in the pathogenesis of severe pancreatitis, as they not only reflect the intensity of the inflammatory process but also serve as indicators of illness progression and potential severity.^[15,19]^

In our study, 192 patients with AP from a single clinical center were retrospectively analyzed. Univariate analysis, least absolute shrinkage and selection operator regression, and binary logistic regression were used sequentially. From a pool of 16 candidate biomarkers, we identified the WBC count, neutrophil count, NLR, and glucose level as independent predictive factors for the early stages of M-SAP/SAP. Their status as independent predictors implies that each marker contributes unique, nonredundant information to the risk assessment model. Specifically, the multivariate analysis demonstrated that for every unit increase in WBC count, neutrophil count, NLR, and glucose level, the odds of developing M-SAP/SAP increased by factors of 1.177, 1.185, 1.053, and 1.154, respectively (all *P* < .05), even after adjusting for other clinical covariates. This independent association underscores that these markers are not merely surrogate indicators of a single underlying process but rather capture distinct pathophysiological dimensions – innate immune activation (WBC/neutrophils), immune dysregulation (NLR), and stress-induced metabolic derangement (glucose) – that collectively contribute to disease progression. We constructed early multi-indicator prediction models with 4 prognostic indices. Through etiological grouping analysis, we found that all 4 inflammatory indices were clearly and statistically significantly correlated with the development of M-SAP/SAP for biliary AP and alcoholic AP patients, and the combination of 4 biomarkers, WBC-neutrophil-NLR-glucose (WNNG) level, performed better than any single factor. For biliary AP and alcoholic AP, we clarified their differences by plotting the ROC curve, and the AUC values were 0.787 and 0.89, respectively, indicating that the model can predict M-SAP/SAP development.

Recent advances in biomarker research have significantly strengthened the prognostic evaluation of AP. For example, Nagy et al^[20]^ demonstrated that hyperglycemia is dose-dependently correlated with AP severity and mortality, with a peak in-hospital glucose level >7 mmol/L serving as a robust predictor of SAP (OR = 14.490, 95% CI = 4.443–47.264) and mortality (OR = 4.750, 95% CI = 1.370–16.476). ROC curve analysis findings further validated the discriminative capacity of this glucose level peak for mortality (AUC = 0.703; cutoff = 6.665 mmol/L) and AP severity (AUC = 0.732; cutoff = 7.355 mmol/L). Chen et al^[21]^ identified systemic inflammatory markers – WBC count and neutrophil count – as predictors of AP complications, demonstrating moderate-to-high accuracy for acute respiratory failure (AUC: 0.729 vs 0.746), sepsis (AUC: 0.722 vs 0.737), infectious pancreatic necrosis (AUC: 0.666 vs 0.675), and acute renal failure (AUC: 0.817 vs 0.824). Complementing these findings, Li et al^[22]^ reported that an NLR >16.64 at admission independently predicted a 3.726-fold increase in mortality risk (95% CI = 1.892–7.334, *P* < .001). While single biomarkers show diagnostic promise, integrated multiparameter models demonstrate superior predictive performance. Notably, Kong et al^[23]^ achieved exceptional discrimination for SAP (AUC = 0.963) through the synergistic combination of heparin-binding protein, C-reactive protein, and procalcitonin levels. This paradigm is further supported by Cho et al^[17]^ who reported that serum interleukin-6 (AUC = 0.755, *P* < .001) and CRP2 (AUC = 0.787, *P* < .001) levels collectively improve early severity stratification, suggesting the utility of these factors as combined diagnostic aids. Despite these advancements, current models remain constrained by their reliance on limited biomarker panels or isolated parameters. Our study addresses this critical gap by systematically integrating multidimensional inflammatory indicators, thereby advancing precision in AP risk stratification.

Compared with patients with pancreatitis caused by other etiologies, HTG-AP patients are more likely to have severe systemic inflammatory response syndrome and poor outcomes.^[24,25]^ Findings from previous studies have indicated a positive correlation between the NLR and the severity of AP.^[22,26–28]^ This finding was confirmed for biliary AP and alcoholic AP in our study. We found that the WBC count, neutrophil count, NLR, and glucose level did not differ between MAP and M-SAP/SAP in HTG-AP patients. These findings suggest that the WBC count, neutrophil count, NLR, and glucose level may fail to assess the severity of inflammation in HTG-AP patients and that other parameters are needed for prediction of inflammation severity. The observed discrepancies likely stem from failure to stratify AP patients according to etiology, differences in eligibility criteria, and differences in sample size, with mechanistic variations across causative subtypes. For example, in the study by Lu et al^[28]^ patients with symptom-to-admission intervals exceeding 72 hours were excluded, whereas we used a 24-hour cutoff in our study. In addition, the relatively small sample size in our study may have affected the statistical power. Emerging evidence from clinical investigations has revealed that the neutrophil percentage, Ca^2+^ level, apolipoprotein A/B ratio, C-reactive protein level, LDH level, nutritional risk index, and presence of peritoneal effusion may serve as independent predictors of HTG-SAP pathogenesis and severity.^[29–31]^

We believe that the results of our study may have even greater predictive diagnostic value and offer better clinical applicability. First, we selected patients who presented with abdominal pain with an onset within 24 hours, and we ensured that blood was drawn within 1 hour and that the blood analysis results were obtained within 6 hours. This strict timeframe helps demonstrate that established biomarkers retain their predictive power within this ultra-early operational window, helping to capture key biomarker changes in the early stages of the disease and thereby increasing the sensitivity and specificity of predictions. Second, we conducted etiological stratification analysis for patients with AP, dividing them into 4 groups: biliary pancreatitis, hyperlipidemic pancreatitis, alcoholic pancreatitis, and idiopathic pancreatitis groups. This stratification method accounted for the potential impact of different etiologies on inflammatory marker levels, thus providing a more accurate predictive model. For example, biliary pancreatitis may be directly related to bacterial infections in the bile ducts, which may affect inflammatory marker levels and patient responses to treatment.^[32,33]^ In contrast, such detailed etiological classifications were not conducted in many other studies, which may lead to less precise treatment and management for patients with AP of different etiologies. Additionally, in clinical practice, cost and operational complexity are key concerns. Measuring the WBC count, neutrophil count, NLR, and glucose level is simple, rapid, and cost-effective, and only collection of a peripheral blood sample is required – a process that is comfortable for patients. These biomarkers are easy to assess, inexpensive, and highly reproducible. Their use not only alleviates the economic burden on patients but also empowers clinicians, particularly those in primary care settings, to better manage AP patients.

This study has several limitations that warrant consideration. First, combining M-SAP and SAP in the analysis may have distorted predictive accuracy and reduced clinical relevance owing to inherent differences in the clinical manifestations and outcomes of these 2 subgroups. While increasing the sample size increased the statistical reliability, this methodological approach may have partially obscured clinically relevant distinctions, thereby affecting the precision and applicability of the findings. Second, as a single-center retrospective study, the generalizability of its findings is limited. This limitation primarily manifests in distinct patient population characteristics – the demographic and clinical features of our cohort may not represent broader populations; unique institutional practices – center-specific treatment protocols, resource allocation, and decision-making culture directly influence intervention implementation and outcome assessment; inherent limitations of retrospective data – potential biases and information gaps diminish the applicability of conclusions to other healthcare settings. Therefore, our findings are most applicable to institutions where patient baseline characteristics, resource allocation, and treatment protocols closely resemble those of our center. Third, while predictive models for biliary and alcoholic AP were developed, prospective validation of these models is required to confirm their accuracy across diverse real-world populations prior to clinical implementation. To address these limitations, 3 directions should be prioritized in future studies: refining patient stratification by integrating individualized biomarkers, dynamic disease progression markers, and etiology-specific risk profiles to enable granular subgroup analyses; conducting multicenter prospective validations to strengthen external validity and ensure consistency across heterogeneous clinical settings; and establishing longitudinal cohorts to capture temporal changes in AP severity, thereby advancing the mechanistic understanding of pathogenesis and enabling personalized prognostic stratification. These strategies will facilitate the translation of research insights into targeted clinical decision-making frameworks.

The practical value of our findings lies in the accessibility and simplicity of the WNNG panel. In emergency and primary care settings where complex scoring systems like APACHE-II or Ranson are impractical, measuring these 4 parameters upon admission provides a rapid, cost-effective, and readily available risk stratification tool. Clinicians can apply the established cutoff values (e.g., NLR > 10.42 for alcoholic AP; WBC > 12.95 × 10^9^/L for biliary AP) to identify high-risk patients who may benefit from immediate transfer to a higher level of care, more frequent vital sign monitoring, early fluid resuscitation, or early nutritional support. Importantly, the etiology-specific performance of this panel should guide its application: NLR may serve as a particularly sensitive trigger for closer monitoring in alcoholic AP, whereas WBC counts may be more useful in biliary cases. In practice, a combined approach – using the panel score to identify high-risk patients, followed by targeted clinical reassessment – offers the greatest potential to improve early triage, resource allocation, and patient outcomes. For instance, a patient with alcoholic AP presenting with an admission NLR >10.42 could be automatically flagged for intensive care unit consultation and hourly vital sign monitoring, whereas a patient with biliary AP and WBC >12.95 × 10^9^/L might warrant early surgical or endoscopic evaluation. This biomarker-directed triage algorithm could be operationalized as a simple bedside checklist in emergency departments. To facilitate clinical adoption, we propose that future studies develop a simple point-based scoring system derived from these 4 biomarkers, which could be integrated into electronic health records for automated risk alerting at the time of admission.

## 5. Conclusion

In this retrospective analysis, the medical records of patients with biliary and alcoholic AP were evaluated to identify independent factors for predicting AP severity and to develop an early prediction model. The model, which incorporates the WBC count, neutrophil count, NLR, and glucose level, accurately predicted AP severity within 24 hours of admission. Each marker independently contributes to the model’s performance, reflecting distinct aspects of the inflammatory and metabolic response. The WNNG panel offers a simple, rapid, and cost-effective tool for early risk stratification in primary and emergency care settings. By identifying patients at high risk for progression to M-SAP/SAP at the time of admission, this model may aid clinicians in making timely triage and treatment decisions, thereby potentially improving outcomes for patients with severe pancreatitis.

## Author contributions

**Data curation:** Zhongshan Li, Yue Lou, Guangfeng Zhang, Xuan Fang, Shiming Chen.

**Methodology:** Zhongshan Li, Shiming Chen.

**Project administration:** Zhongshan Li, Shiming Chen.

**Software:** Zhongshan Li, Shiming Chen.

**Conceptualization:** Yue Lou.

**Investigation:** Yue Lou, Shiming Chen.

**Resources:** Guangfeng Zhang, Xuan Fang, Shiming Chen.

**Formal analysis:** Shiming Chen.

**Writing – original draft:** Zhongshan Li, Shiming Chen.

**Writing – review & editing:** Shiming Chen.

## References

[1] Mederos MA Reber HA Girgis MD. Acute pancreatitis: a review. JAMA. 2021;325:382–90. 33496779 10.1001/jama.2020.20317

[2] Leppäniemi A Tolonen M Tarasconi A. 2019 WSES guidelines for the management of severe acute pancreatitis. World J Emerg Surg. 2019;14:27. 31210778 10.1186/s13017-019-0247-0 PMC6567462

[3] Boxhoorn L Voermans RP Bouwense SA. Acute pancreatitis. Lancet. 2020;396:726–34. 32891214 10.1016/S0140-6736(20)31310-6

[4] Chen X Lin Z Chen Y Lin C. C-reactive protein/lymphocyte ratio as a prognostic biomarker in acute pancreatitis: a cross-sectional study assessing disease severity. Int J Surg. 2024;110:3223–9. 38446844 10.1097/JS9.0000000000001273 PMC11175793

[5] Szatmary P Grammatikopoulos T Cai W. Acute pancreatitis: diagnosis and treatment. Drugs. 2022;82:1251–76. 36074322 10.1007/s40265-022-01766-4 PMC9454414

[6] Staubli SM Oertli D Nebiker CA. Laboratory markers predicting severity of acute pancreatitis. Crit Rev Clin Lab Sci. 2015;52:273–83. 26173077 10.3109/10408363.2015.1051659

[7] Balthazar EJ. Acute pancreatitis: assessment of severity with clinical and CT evaluation. Radiology. 2002;223:603–13. 12034923 10.1148/radiol.2233010680

[8] Ranson JH Rifkind KM Roses DF Fink SD Eng K Spencer FC. Prognostic signs and the role of operative management in acute pancreatitis. Surg Gynecol Obstet. 1974;139:69–81. 4834279

[9] Knaus WA Draper EA Wagner DP Zimmerman JE. APACHE II: a severity of disease classification system. Crit Care Med. 1985;13:818–29. 3928249

[10] Papachristou GI Muddana V Yadav D. Comparison of BISAP, Ranson’s, APACHE-II, and CTSI scores in predicting organ failure, complications, and mortality in acute pancreatitis. Am J Gastroenterol. 2010;105:435–41; quiz 442. 19861954 10.1038/ajg.2009.622

[11] Wu BU Johannes RS Sun X Tabak Y Conwell DL Banks PA. The early prediction of mortality in acute pancreatitis: a large population-based study. Gut. 2008;57:1698–703. 18519429 10.1136/gut.2008.152702

[12] Mortele KJ Wiesner W Intriere L. A modified CT severity index for evaluating acute pancreatitis: improved correlation with patient outcome. AJR Am J Roentgenol. 2004;183:1261–5. 15505289 10.2214/ajr.183.5.1831261

[13] Sahu B Abbey P Anand R Kumar A Tomer S Malik E. Severity assessment of acute pancreatitis using CT severity index and modified CT severity index: correlation with clinical outcomes and severity grading as per the Revised Atlanta Classification. Indian J Radiol Imaging. 2017;27:152–60. 28744075 10.4103/ijri.IJRI_300_16 PMC5510312

[14] Banks PA Bollen TL Dervenis C. Classification of acute pancreatitis – 2012: revision of the Atlanta classification and definitions by international consensus. Gut. 2013;62:102–11. 23100216 10.1136/gutjnl-2012-302779

[15] Behera M Mishra D Sahu M. C-reactive protein/albumin and ferritin as predictive markers for severity and mortality in patients with acute pancreatitis. Gastroenterol Rev. 2023;18:168–74. 10.5114/pg.2022.115609 PMC10395065 37538281

[16] Carrascal M Areny-Balagueró A de-Madaria E. Inflammatory capacity of exosomes released in the early stages of acute pancreatitis predicts the severity of the disease. J Pathol. 2021;256:83–92. 34599510 10.1002/path.5811

[17] Cho IR Do MY Han SY Jang SI Cho JH. Comparison of interleukin-6, C-reactive protein, procalcitonin, and the computed tomography severity index for early prediction of severity of acute pancreatitis. Gut Liver. 2023;17:629–37. 36789576 10.5009/gnl220356 PMC10352050

[18] He SS Li D He QY. Establishment of early multi-indicator prediction models of moderately severe acute pancreatitis and severe acute pancreatitis. Gastroenterol Res Pract. 2022;2022:5142473. 35419053 10.1155/2022/5142473 PMC9001090

[19] Wei X Guo S Wang Q. Predictive value of troponin I, creatinine kinase isoenzyme and the new Japanese severity score in severe acute pancreatitis. Patient Prefer Adherence. 2024;18:1131–40. 38863946 10.2147/PPA.S462244 PMC11164687

[20] Nagy A Juhász MF Görbe A. Glucose levels show independent and dose-dependent association with worsening acute pancreatitis outcomes: post-hoc analysis of a prospective, international cohort of 2250 acute pancreatitis cases. Pancreatology. 2021;21:1237–46. 34332908 10.1016/j.pan.2021.06.003

[21] Chen X Ning J Li Q Kuang W Jiang H Qin S. Prediction of acute pancreatitis complications using routine blood parameters during early admission. Immun Inflammation Dis. 2022;10:e747. 10.1002/iid3.747 PMC9695081 36444624

[22] Li Y Zhao Y Feng L Guo R. Comparison of the prognostic values of inflammation markers in patients with acute pancreatitis: a retrospective cohort study. BMJ Open. 2017;7:e013206. 10.1136/bmjopen-2016-013206 PMC5372142 28348184

[23] Kong D Lei Z Wang Z. A novel HCP (heparin-binding protein-C reactive protein-procalcitonin) inflammatory composite model can predict severe acute pancreatitis. Sci Rep. 2023;13:9440. 37296194 10.1038/s41598-023-36552-z PMC10256784

[24] Xu M-S Xu J-L Gao X. Clinical study of neutrophil-to-lymphocyte ratio and platelet-to-lymphocyte ratio in hypertriglyceridemia-induced acute pancreatitis and acute biliary pancreatitis with persistent organ failure. World J Gastrointest Surg. 2024;16:1647–59. 38983313 10.4240/wjgs.v16.i6.1647 PMC11230014

[25] Zhu Y Pan X Zeng H. A study on the etiology, severity, and mortality of 3260 patients with acute pancreatitis according to the Revised Atlanta Classification in Jiangxi, China over an 8-year period. Pancreas. 2017;46:504–9. 28196012 10.1097/MPA.0000000000000776

[26] Abayli B Gençdal G Değirmencioğlu S. Correlation between neutrophil/lymphocyte ratio and Ranson score in acute pancreatitis. J Clin Lab Anal. 2018;32:e22437. 29575044 10.1002/jcla.22437 PMC6816873

[27] Huang L Chen C Yang L Wan R Hu G. Neutrophil-to-lymphocyte ratio can specifically predict the severity of hypertriglyceridemia-induced acute pancreatitis compared with white blood cell. J Clin Lab Anal. 2019;33:e22839. 30737845 10.1002/jcla.22839 PMC6528595

[28] Lu Z Chen X Ge H. Neutrophil-lymphocyte ratio in patients with hypertriglyceridemic pancreatitis predicts persistent organ failure. Gastroenterol Res Pract. 2022;2022:8333794. 35340692 10.1155/2022/8333794 PMC8942680

[29] Hu YQ Tao X Wu HB. Predicting severity in hypertriglyceridemia-induced acute pancreatitis: the role of neutrophils, calcium, and apolipoproteins. Med Sci Monit. 2024;30:e942832. 38321725 10.12659/MSM.942832 PMC10860425

[30] Shuanglian Y Huiling Z Xunting L. Establishment and validation of early prediction model for hypertriglyceridemic severe acute pancreatitis. Lipids Health Dis. 2023;22:218. 38066493 10.1186/s12944-023-01984-z PMC10709974

[31] Wang YZ Yun YL Ye T Yao WT Guo YF Huang LY. Value of the systemic immunoinflammatory index, nutritional risk index, and triglyceride-glucose index in predicting the condition and prognosis of patients with hypertriglyceridemia-associated acute pancreatitis. Front Nutr. 2025;12:1523046. 39949545 10.3389/fnut.2025.1523046 PMC11821461

[32] Baron TH DiMaio CJ Wang AY Morgan KA. American Gastroenterological Association clinical practice update: management of pancreatic necrosis. Gastroenterology. 2020;158:67–75.e1. 31479658 10.1053/j.gastro.2019.07.064

[33] van Geenen EJ van der Peet DL Bhagirath P Mulder CJJ Bruno MJ. Etiology and diagnosis of acute biliary pancreatitis. Nat Rev Gastroenterol Hepatol. 2010;7:495–502. 20703238 10.1038/nrgastro.2010.114

